# Current Status and Associated Factors of Discharge Readiness in Ambulatory‐Surgery Patients Based on Transition Theory: A Cross‐Sectional Study

**DOI:** 10.1002/nop2.70622

**Published:** 2026-06-06

**Authors:** Jing Zhang, Xiaoli Xing, Xiaodan Xia, Enjie Ping, Xiufang Zhang, Jingjing Liu, Xiaorong Luan

**Affiliations:** ^1^ Department of Nursing The First Hospital of Anhui University of Science and Technology Huainan China; ^2^ Department of Daytime Medical Center The First Hospital of Anhui University of Science and Technology Huainan China; ^3^ Department of Hospital Infection Management Qilu Hospital of Shandong University Jinan China

**Keywords:** ambulatory surgery, discharge readiness, family Apgar index, health literacy, quality of discharge teaching

## Abstract

**Aims:**

This study aimed to identify the discharge readiness level of ambulatory surgery patients and examine the effects of these variables on the discharge readiness of ambulatory surgery patients.

**Design:**

A cross‐sectional quantitative design was used.

**Methods:**

A convenience sample of 212 patients undergoing ambulatory surgery between January 2022 and June 2023 was enrolled. Discharge readiness, quality of discharge teaching, health literacy and family support were measured. Pearson correlation and multiple linear regression were used to examine associations and explanatory factors.

**Results:**

It was determined that the discharge readiness of patients with ambulatory surgery was at a medium level (91.10 ± 8.53). Discharge readiness was positively associated with quality of discharge teaching (*r* = 0.703, *p* < 0.01), health literacy (*r* = 0.503, *p* < 0.01) and family support (*r* = 0.305, *p* < 0.01). Stepwise multiple linear regression showed that quality of discharge teaching (*β* = 0.613, *p* < 0.001), health literacy (*β* = 0.205, *p* < 0.001), surgical specialty (ophthalmology: *β* = 0.370, *p* < 0.001; otorhinolaryngology: *β* = 0.138, *p* = 0.002), age groups (45–59 years: *β* = −0.106, *p =* 0.012; 75–89 years: *β* = −0.175, *p* < 0.001), family support (*β* = 0.112, *p* = 0.007), living arrangement (*β* = 0.101, *p* = 0.011) and occupation (retirement: *β* = −0.095, *p* = 0.041) were significantly associated with discharge readiness. The final model was statistically significant (adjusted *R* ^2^ = 0.682), explaining 68.2% of the variance in discharge readiness.

**Conclusion:**

Discharge readiness of ambulatory surgery patients was moderate. The quality of discharge teaching, health literacy, surgical specialty (ophthalmology, otorhinolaryngology), age groups (45–59 and 75–89 years), family support, living arrangement and occupation (retirement), which cover multiple dimensions such as spanning personal, environmental, family and therapeutic factors, were associated with discharge readiness.

**Patient or Public Contribution:**

Data were collected via questionnaire from participants. Findings can inform personalised discharge preparation programs to improve the discharge readiness of day surgery patients.

**Reporting Method:**

This study was reported in accordance with the STROBE guidelines.

## 1 Introduction

Ambulatory surgery refers to admission, procedure and discharge within 24 h, extended to 48 h only in special cases (Bailey et al. 2019). This approach effectively utilises healthcare resources, shortens the care trajectory, reduces in‐hospital waiting time and alleviates patients' financial burden (Zhang and Luan 2023; Guo et al. 2024). However, the abbreviated length of stay leaves patients with unmet nursing needs at discharge, especially those undergoing Grade III–IV procedures, which may lead to insufficient discharge readiness and hinder recovery.

Clinical studies have indicated that inadequate discharge readiness in ambulatory surgery patients leads to a series of adverse clinical outcomes. First, the incidence of postoperative complications is significantly elevated; for instance, the occurrence rates of wound infection, haemorrhage, poor pain control, electrolyte disturbance and other complications are markedly higher in patients with inadequate discharge readiness compared to those with sufficient discharge readiness (Zhang et al. 2022). Second, the rate of unplanned readmission in patients increases in the short term, accompanied by a heightened risk of readmission (Shi et al. 2022). Third, due to the lack of knowledge regarding disease management and self‐care, patients are prone to medication errors, irregular rehabilitation exercises and other problems, which delay the postoperative recovery process and affect the effectiveness of postoperative recovery as well as nursing satisfaction (Xiao et al. 2024; Qiu et al. 2019). Fourth, from the perspective of the rational utilisation of medical resources, inadequate discharge readiness results in the repeated consumption of medical resources, leading to an increase in patients' additional medical expenses and a negative impact on their medical experience (Wang, Liu, and Sheng 2024).

At present, the operational definition of discharge readiness in academia is mainly based on the assessment of patients' actual care needs and abilities. Galvin defined discharge readiness as follows: the comprehensive ability of patients to safely and independently return to home or community settings at discharge and effectively manage health problems after discharge, which specifically encompasses five core dimensions: stability of physical status, cognitive level of the disease and treatment plan, mastery of self‐care skills, ability to utilise social support resources and confidence in coping with sudden health problems (Galvin et al. 2017).

Based on Meleis' Transition Theory, discharge readiness involves clinicians' comprehensive assessment of patients' physical, psychological and social status during the transition back to home and community, thereby determining their capacity for further recovery (Galvin et al. 2017). Higher discharge readiness is associated with lower rates of readmission, mortality and emergency encounters (Kaya et al. 2018). Transition theory posits that the discharge‐to‐recovery trajectory encompasses four interactive dimensions—nature, conditions, nursing therapeutics and response patterns—requiring a holistic, systems‐oriented approach to convalescence (Wang, Li, et al. 2024). Prior correlates factors research has focused on demographic and disease‐specific variables while neglecting transitional care needs and an integrated recovery paradigm. Moreover, studies concentrate on chronic conditions; ambulatory‐surgery readiness—especially for grade III–IV procedures—remains under‐explored.

Nurses play a core role in discharge education, transitional care and readiness assessment. While the Canadian Health Outcomes for Better Information and Care (C‐HOBIC) tool was not directly implemented in this study, our assessment framework demonstrates conceptual alignment with its core principles. C‐HOBIC emphasises capturing patient self‐care capacity and care transition readiness through validated metrics, mirroring the focus of Meleis Transition Theory on adaptive behaviors and health information comprehension during post‐discharge transitions (Hannah et al. 2012; Hreńczuk et al. 2025).

**FIGURE 1:**
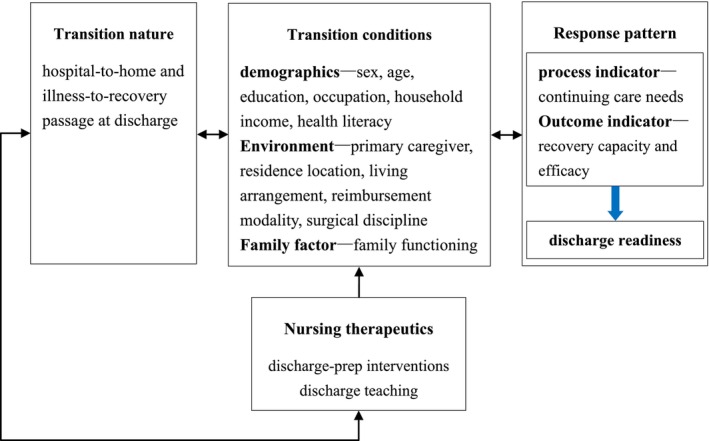
Theoretical framework of this study.

Therefore, using Meleis' Transition Theory and a systems view, we dissect the associated factors of discharge readiness in ambulatory surgery patients across transition nature, conditions, nursing therapeutics and response patterns, aiming to provide reference and basis for formulating personalised discharge preparation service plans and continuing care measures. The theoretical framework of this study is illustrated in Figure 1.

## 2 Methods

### 2.1 Design

A cross‐sectional and descriptive quantitative design was used, reported according to STROBE guidelines (STROBE Initiative 2007).

### 2.2 Study Participants

Convenience sampling was used to enrol patients undergoing grade III—IV ambulatory surgery at the Ambulatory Surgery Center of a university affiliated hospital between January 2022 and June 2023.

### 2.3 Sample Size Calculation

Sample size was calculated using G*Power 3.1.9.7 for multiple linear regression. 18 potential predictors were selected based on the Transition Theory framework (personal, environmental, family, therapeutic and clinical variables). We calculated the required sample. With medium effect size ƒ^2^ = 0.15, *α* = 0.05, power = 0.80, the required sample was 150. Allowing 20% attrition, the target sample was 188. A total of 220 questionnaires were distributed; 8 were invalid, leaving 212 valid cases (96.4% valid rate), meeting the required sample size.

### 2.4 Inclusion and Exclusion Criteria

Inclusion criteria: (1) Patients judged suitable for ambulatory surgery after systematic assessment and who successfully completed the procedure; (2) age ≥ 18 years with normal comprehension and communication and (3) informed consent and voluntary participation. Exclusion criteria: (1) Conversion to inpatient; (2) severe organ dysfunction and (3) inability to cooperate with the survey.

### 2.5 Instruments

This study used the ‘Readiness for Hospital Discharge Scale’ to assess the discharge readiness of patients with ambulatory surgery. The data of patients were collected using the ‘Readiness for Hospital Discharge Scale’, ‘All Aspects of Health Literacy Scale’ and ‘Family APGAR Index’. In addition, all participants also used the ‘General Information Form’ regarding sociodemographic and surgical characteristics.

#### 2.5.1 General Information Form

Based on a literature review and the study objectives, we developed a general information questionnaire covering age (years), sex, education, occupation, marital status, living arrangement, residence, primary caregiver, household income, medical expense reimbursement type and surgical specialty.

#### 2.5.2 Readiness for Hospital Discharge Scale (RHDS)

We used the Chinese version translated and revised by Lin (Lin et al. 2014). It comprises three dimensions—personal status, adaptive capacity and expected support—containing 12 items scored 0–10. Higher scores indicate greater readiness. Mean item scores ≥ 9 denote very high readiness, 8–8.99 high, 7–7.99 moderate and < 7 low. Cronbach's *α* was 0.89 in the original and 0.90 in this study.

#### 2.5.3 Quality of Discharge Teaching Scale (QDTS)

Translated and revised by Wang et al. (2016), it includes three dimensions—content needed, content received and teaching skills/effectiveness—each item scored 0–10. The total score is the sum of the latter two dimensions; higher scores indicate better quality (Wang, Li, et al. 2024). Cronbach's *α* was 0.92 originally and 0.89 in this study.

#### 2.5.4 All Aspects of Health Literacy Scale (AAHLS)

This scale was developed by Chinn et al. (Chinn and McCarthy 2013), and translated into Chinese by Wu et al. (2017). It contains 11 items across three dimensions—ability to use written health information, communicate with providers and evaluate/apply health information—each scored 1–3; higher scores indicate higher health literacy. Cronbach's *α* was 0.81 originally and 0.79 here.

#### 2.5.5 Family APGAR Index

This scale was developed by Smilkstein (1978) and translated into Chinese by Lv and Gu (1995). It captures individual subjective perceptions of family functioning and satisfaction. It comprises five items—adaptation, partnership, growth, affection and resolve—each scored 0–2, yielding a total of 10 points; higher scores indicate better family function. The index is widely used in clinical research and demonstrates good reliability and validity. Cronbach's *α* was 0.73 in this survey.

### 2.6 Procedure

A pilot study with 50 patients was conducted to verify the feasibility of the questionnaire. Researchers used standardised scripts to explain the study's purpose, obtain informed consent and emphasise voluntary participation and the right to withdraw without affecting care. Data were confidential and anonymous. To shorten completion time and improve quality, the survey was split into two sessions. Session 1 (post‐admission, pre‐surgery): general information, health literacy, family function. Session 2 (pre‐discharge, post‐surgery): discharge‐readiness and discharge‐teaching quality. Forms were checked for completeness and collected on site. Double data entry, cleaning and analysis followed. A total of 220 questionnaires were distributed; 8 were excluded due to excessive missing items or incomplete responses. Ultimately, a total of 212 valid responses were collected (response rate 96.4%). Item‐level missing data in valid questionnaires were handled by listwise deletion.

### 2.7 Statistical Analysis

SPSS 26.0 was used for data analyses. Normality was tested using the Shapiro–Wilk test. Continuous variables with a normal distribution were expressed as mean ± SD, while variables violating normality were expressed as median (*P*_25_, *P*_75_). Between‐group comparisons were performed using independent‐samples *t*‐test, one‐way ANOVA, or Kruskal–Wallis *H* test as appropriate. Pearson correlation was used for the main analysis, and Spearman's rho was performed for sensitivity analysis. Multiple linear regression was performed using the stepwise method; variables were entered into the model based on statistical significance (from univariate analysis and correlation analysis) and theoretical relevance derived from Meleis' Transition Theory. Variance inflation factor (VIF) was used to detect multicollinearity. *p* < 0.05 was considered statistically significant. Visualisations were created with the ‘corrplot’ package in R 4.5.1. Missing data were handled by listwise deletion. Missingness was considered missing completely at random (MCAR).

### 2.8 Ethics Approval and Consent to Participate

This study was conducted in accordance with the Declaration of Helsinki and approved by the ethics committee of The First Hospital of Anhui University of Science and Technology (Number: 2022‐YJ‐004‐01). Written informed consent was obtained from all participants.

## 3 Results

### 3.1 Sample Characteristics

In this study, patients were aged 18–89 years. According to the standards of the World Health Organization (WHO), the age was divided into four groups: youth (18–44 years), middle‐aged (45–59 years), young‐old (60–74 years) and old (75–89 years) (Hu and Xiao 2022). The sample included 124 males (58.5%) and 88 females (41.5%). Four surgical departments were included: general surgery (laparoscopic cholecystectomy, laparoscopic inguinal herniorrhaphy, thyroidectomy, radical operation for perianal abscess), orthopaedics (arthroscopic knee joint surgery, arthroscopic shoulder joint surgery, carpal tunnel release, cubital tunnel release, removal of internal fixation of femur), ophthalmology (cataract surgery, intravitreal drug injection, vitrectomy) and otorhinolaryngology (endoscopic sinus surgery, nasal polypectomy, endoscopic epiglottic lesion resection, endoscopic vocal cord surgery, endoscopic laryngeal surgery). There were no significant differences in age, gender or surgical specialty between respondents and excluded participants. Other sociodemographic and clinical characteristics of patients were shown in Table 1.

**TABLE 1:** General data characteristics of ambulatory surgery patients (*N* = 212).

Variables	Category	*N*	%
Gender	Male	124	58.5
	Female	88	41.5
Age (years)	18–44	84	39.6
	45–59	73	34.4
	60–74	37	17.5
	75–89	18	8.5
Education	≤ Primary	49	23.1
	Junior high	68	32.1
	Senior/technical	32	15.1
	College	32	15.1
	≥ Bachelor	31	14.6
Occupation	Farmer	34	16.0
	Worker	40	18.9
	Civil servant/technician	27	12.7
	Self‐employed	26	12.3
	Unemployed	40	18.9
	Retired	45	21.2
Marital status	Married	178	84.0
	Unmarried/divorced/widowed	34	16.0
Living arrangement	Alone	25	11.8
	Not alone	187	88.2
Primary caregiver	Self	140	66.0
	Spouse/parents/children	72	34.0
Residence	City	149	70.3
	Countryside	63	29.7
Household monthly per capita income (¥)	≤ 3000	86	40.6
	3001–5000	103	48.6
	≥ 5001	23	10.8
Medical reimbursement	Employee insurance	89	42.0
	Resident insurance	107	50.5
	Self‐pay	16	7.5
Surgical specialty	Ophthalmology	63	29.7
	Otorhinolaryngology	59	27.8
	Orthopaedics	25	11.8
	General surgery	65	30.7

*Note:* General surgery included laparoscopic cholecystectomy, laparoscopic inguinal herniorrhaphy, thyroidectomy and radical operation for perianal abscess.

### 3.2 Current Status of Discharge Readiness

The mean RHDS score was (91.10 ± 8.53), item mean (7.59 ± 0.71), indicating a moderate level. An analysis of the three dimensions of the RHDS revealed the following: the score for the personal status was (22.98 ± 2.82) with a mean item score of (7.66 ± 0.94); the score for the adaptive capacity was (37.31 ± 4.28) with a mean item score of (7.46 ± 0.86) and the score for the expected support was (30.81 ± 2.94) with a mean item score of (7.70 ± 0.73). In this study, 36 (17.0%) patients showed low readiness, 117 (55.2%) moderate, 51 (24.1%) high and 8 (3.8%) very high readiness.

### 3.3 Univariate Analysis of Discharge Readiness

Univariate analyses showed significant differences in RHDS by age (years), education, occupation, living arrangement, primary caregiver, residence, household income, reimbursement type and surgical specialty (all *p* < 0.05) (Table 2).

**TABLE 2:** Univariate analysis of RHDS in ambulatory‐surgery patients (*N* = 212).

Variable	Category	*N* (%)	RHDS $\bar{x}\pm s$/*M* (*P*_25_, *P*_75_)	Statistic	*p*
Gender	Male	124 (58.5)	91.95 ± 8.71	*t =* 1.736	0.084
	Female	88 (41.5)	89.90 ± 8.18		
Age (years)	18–44	84 (39.6)	93.00(90.00,98.00)	*H* = 15.487	0.001
	45–59	73 (34.4)	89.00(85.00,95.00)		
	60–74	37 (17.5)	92.00(85.50,96.00)		
	75–89	18 (8.5)	84.50(74.75,94.50)		
Education	≤ Primary	49 (23.1)	87.00 ± 7.00	*F* = 10.344	< 0.001
	Junior high	68 (32.1)	89.90 ± 8.79		
	Senior/technical	32 (15.1)	90.84 ± 7.44		
	College	32 (15.1)	93.59 ± 8.39		
	≥ Bachelor	31 (14.6)	97.90 ± 6.76		
Occupation	Farmer	34 (16.0)	87.38 ± 7.45	*F* = 6.094	< 0.001
	Worker	40 (18.9)	93.45 ± 8.07		
	Civil servant/technician	27 (12.7)	97.48 ± 6.07		
	Self‐employed	26 (12.3)	89.85 ± 7.04		
	Unemployed	40 (18.9)	90.23 ± 8.40		
	Retired	45 (21.2)	89.49 ± 9.60		
Marital status	Married	178 (84.0)	91.04 ± 8.27	*t =* 0.211	0.833
	Unmarried/divorced/widowed	34 (16.0)	91.38 ± 9.93		
Living arrangement	Alone	25 (11.8)	87.00 ± 8.78	*t =* −2.593	0.010
	Not alone	187 (88.2)	91.65 ± 8.37		
Primary caregiver	Self	140(66.0)	92.65 ± 8.03	*t =* 3.808	< 0.001
	Spouse/parents/children	72 (34.0)	88.08 ± 8.72		
Residence	City	149 (70.3)	92.46 ± 8.45	*t =* 3.666	< 0.001
	Countryside	63 (29.7)	87.89 ± 7.90		
Household monthly per capita income (¥)	≤ 3000	86 (40.6)	88.09 ± 8.42	*F* = 10.798	< 0.001
	3001–5000	103 (48.6)	92.67 ± 7.40		
	≥ 5001	23 (10.8)	95.30 ± 10.22		
Medical reimbursement	Employee insurance	89 (42.0)	93.47 ± 8.83	*F* = 6.409	0.002
	Resident insurance	107 (50.5)	89.55 ± 8.06		
	Self‐pay	16 (7.5)	88.25 ± 6.91		
Surgical specialty	Ophthalmology	63 (29.7)	92.68 ± 9.53	*F* = 5.275	0.002
	Otorhinolaryngology	59 (27.8)	91.61 ± 6.82		
	Orthopaedics	25 (11.8)	85.08 ± 9.76		
	General surgery	65 (30.7)	91.42 ± 7.56		

*Note: t*‐test; one‐way ANOVA; Kruskal–Wallis *H* test.

### 3.4 Discharge Readiness Correlates With Teaching Quality, Health Literacy and Family Support

**FIGURE 2:**
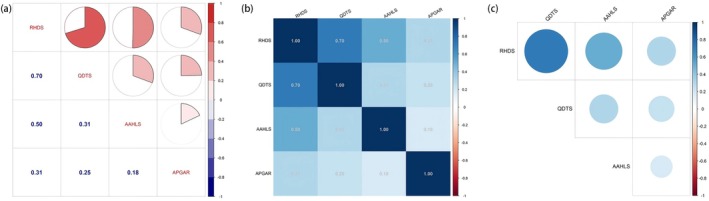
Correlation heatmap of RHDS with (a–c) QDTS, AAHLS and APGAR in ambulatory‐surgery patients.

Discharge readiness was positively correlated with QDTS, AAHLS and APGAR (all *p* < 0.01). Spearman's rho sensitivity analysis yielded consistent results (Table 3). A correlation heatmap is shown in Figure 2.

**TABLE 3:** Correlations between RHDS and QDTS, AAHLS and APGAR (*r*).

Variable	RHDS	Personal status	Adaptive capacity	Expected support
QDTS	0.703**	0.610**	0.632**	0.536**
Content needed	0.304**	0.330**	0.304**	0.125
Content received	0.387**	0.410**	0.383**	0.172*
Teaching skills/effect	0.704**	0.571**	0.614**	0.604**
AAHLS	0.503**	0.381**	0.459**	0.428**
Ability to use written health information	0.255**	0.165*	0.235**	0.239**
Communication with providers	0.488**	0.412**	0.413**	0.420**
Evaluate/apply health information	0.425**	0.303**	0.411**	0.345**
APGAR score	0.305**	0.262**	0.227**	0.305**

*Note:* Pearson correlation was used for main analysis. Spearman's rho was performed for sensitivity analysis and yielded consistent directional trends.

* *p <* 0.05 (two‐tailed).

** *p <* 0.01.

### 3.5 Multiple Linear Regression Analysis of Discharge Readiness

Stepwise multiple linear regression was conducted with the total RHDS score as the dependent variable. Variables that were significant in univariate and correlation analyses were included as independent variables. Age was treated as a categorical variable with dummy coding (reference: 18–44 years). The final model included quality of discharge teaching, ophthalmology, health literacy, age (75–89), otorhinolaryngology, family APGAR score, living arrangement, age (45–59) and occupation (retirement). The coding scheme for independent variables and dummy variables was shown in Table 4.

**TABLE 4:** Coding scheme for independent variables (dummy coding).

Variables	Coding
Age (years)	Reference: 18–44; Dummy1 (45 –59 = 1), Dummy2 (60 –74 = 1), Dummy3 (75 –89 = 1)
Education	1 = Primary or below; 2 = Junior high; 3 = Senior/Technical; 4 = College; 5 ≥ Bachelor
Occupation	Reference: Unemployed; Dummy1 (Farmer = 1), Dummy2 (Worker = 1), Dummy3 (Civil servant/technician = 1), Dummy4 (Self‐employed = 1), Dummy5 (Retired = 1)
Living arrangement	1 = Alone; 2 = Not alone
Primary caregiver	1 = Self; 2 = Spouse/Parents/Children
Residence	1 = City; 2 = Countryside
Household monthly per capita income (¥)	1 = ≤ 3000; 2 = 3001–5000; 3 = ≥ 5001
Medical reimbursement	Reference: Self‐pay; Dummy1 (Employee insurance = 1), Dummy2 (Resident insurance = 1)
Surgical specialty	Reference: General surgery; Dummy1 (Ophthalmology = 1), Dummy2 (Otorhinolaryngology = 1), Dummy3 (Orthopedics = 1)
QDTS	Continuous
AAHLS	Continuous
APGAR	Continuous

The model was statistically significant (*F* = 51.211, *p* < 0.001). The adjusted *R* ^2^ was 0.682, indicating that these variables explained 68.2% of the total variance in discharge readiness. All VIFs values were less than 2.0, suggesting no multicollinearity. Sensitivity analyses using Spearman's rho and categorical age confirmed the robustness of the main results. The detailed results were presented in Table 5.

**TABLE 5:** Multiple linear regression for factors associated with discharge readiness in ambulatory‐surgery patients (*N* = 212).

Variable	*B*	SE	95% CI for *B*	*β*	*t*	*p*	VIF
Constant	7.867	4.507	−1.020, 16.753	—	1.746	0.082	
QDTS	0.451	0.033	0.385, 0.517	0.613	13.531	< 0.001	1.361
Ophthalmology	6.897	0.978	4.968, 8.825	0.370	7.052	< 0.001	1.828
AAHLS	0.520	0.119	0.286, 0.755	0.205	4.380	< 0.001	1.455
Age (75–89)	−5.355	1.369	−8.054, −2.657	−0.175	−3.913	< 0.001	1.332
Otorhinolaryngology	2.626	0.836	0.978, 4.273	0.138	3.142	0.002	1.284
APGAR	0.602	0.222	0.165, 1.039	0.112	2.717	0.007	1.134
Living arrangement	2.671	1.046	0.609, 4.733	0.101	2.554	0.011	1.041
Age (45–59)	−1.902	0.747	−3.374, −0.430	−0.106	−2.548	0.012	1.152
Occupation (retirement)	−1.986	0.965	−3.890, −0.083	−0.095	−2.058	0.041	1.426

*Note:* Model statistics: *F* = 51.211, *p* < 0.001; Adjusted *R* ^2^ = 0.682.

Reference group for age: 18–44 years; reference group for surgery: general surgery.

All VIF < 2.0, indicating no multicollinearity.

## 4 Discussion

### 4.1 Moderate Discharge Readiness Among Ambulatory‐Surgery Patients

Discharge readiness was moderate, consistent with previous literature (Qiu et al. 2019; Yang et al. 2025). In this study, 36 patients (17%) scored below 7 on the average item score for discharge readiness, indicating insufficient preparedness. This may be attributable to the high proportion of older adults (128, 60.4%) with lower education, limited comprehension and reduced problem‐solving capacity, thereby reducing overall discharge readiness. Ophthalmic and orthopaedic patients exhibited the greatest deficits, likely because visual or motor impairment heightened concerns about post‐operative self‐care.

The adaptive capacity dimension scored lowest, encompassing self‐care ability, knowledge of self‐care tasks, management of daily needs and execution of home‐based medical care, indicating deficits in requisite knowledge and skills. The underlying reason may be the novelty of ambulatory surgery itself: the markedly shortened inpatient stay leaves patients uncertain about their ability to master the required self‐care knowledge and skills, resulting in diminished adaptive capacity. Therefore, clinicians and nurses should proactively assess adaptive needs and provide targeted, practical discharge education to enhance self‐care knowledge and skills.

### 4.2 Factors Associated With Discharge Readiness

In the present study, stepwise multiple linear regression showed that quality of discharge teaching, health literacy, ophthalmology, otorhinolaryngology, age (45–59), age (75–89), family support, living arrangement and occupation (retirement) were independent factors associated with discharge readiness. The final model explained 68.2% of the total variance, indicating good model fitness.

#### 4.2.1 Quality of Discharge Teaching

It was the strongest explanatory factor, consistent with Zhao et al. (2020) and Qiu et al. (2023). Notably, ‘content needed’ (44.97 ± 3.80) exceeded ‘content received’ (43.79 ± 4.85), indicating unmet educational needs and room for improvement, as also reported by Guo et al. (2025). Patients' actual uptake of discharge education is influenced by two key dimensions: (a) individual receptivity, comprehension and learning capacity—those with stronger abilities assimilate and apply self‐care instructions effectively, whereas limited capacity results in poor retention and (b) variability in nurses' expertise, which produces inconsistent teaching quality. Consequently, standardised training, continuous professional development, real‐time feedback loops and rigorous quality‐control measures are imperative to optimise discharge‐teaching precision and impact.

#### 4.2.2 Health Literacy

The total AAHLS score was moderately and positively associated with overall discharge readiness (*r* = 0.496, *p* < 0.01); higher literacy predicted higher readiness, consistent with Wallace et al. (2016). Higher health literacy strengthens learning, information retrieval, application and appraisal skills, leading to better comprehension of discharge instructions and mastery of self‐care techniques. Motivated to improve their health, these individuals actively seek health knowledge and adopt preventive measures, thereby enhancing discharge readiness.

#### 4.2.3 Surgical Specialty

It was independently associated with discharge readiness. In the multiple linear regression model, patients from ophthalmology and otorhinolaryngology showed significantly higher discharge readiness compared with those from general surgery. In descriptive analysis, ophthalmology patients scored the highest (92.68 ± 9.53) and orthopaedic patients scored the lowest (85.08 ± 9.76). The possible reasons are that unilateral eye surgery and otorhinolaryngology procedures have relatively minor trauma and minimal influence on daily activities, whereas orthopaedic procedures often limit mobility and self‐care ability, which is consistent with studies by Qiu et al. (2023) and Li, Guo, et al. (2024). Since different surgical specialties have distinct characteristics and impose varying degrees of postoperative functional limitation, individualised discharge instructions tailored to each specialty are essential to further improving patients’ discharge readiness.

#### 4.2.4 Age Groups

Readiness scores differed significantly across age groups. Patients aged 45–59 and 75–89 years exhibited lower discharge readiness than those aged 18–44 years. Advancing age independently predicted lower readiness, corroborating Li, Moreira, et al. (2024) and Huang and Peng (2022). The gradual deterioration of age‐related physiological functions diminishes self‐care ability, mobility and memory, thereby impairing personal status and adaptive capacity. Consequently, nurses should reinforce discharge instructions repeatedly for older patients to enhance both comprehension and retention, ultimately improving readiness.

#### 4.2.5 Family Function

The APGAR score reflects family functioning; optimal family dynamics foster emotional exchange among members and mutual support during stressful events. Our findings revealed that 68.9% of ambulatory‐surgery patients reported adequate family functioning, and APGAR was positively associated with overall discharge readiness (*r* = 0.305, *p* < 0.01); higher family support predicted greater readiness. Because patients are not fully recovered upon discharge, continued familial assistance is essential; robust family functioning enhances perceived care, emotional reassurance and practical help. Therefore, clinical doctors and nurses should adopt a family‐centred approach in discharge planning, educating relatives to provide practical, emotional and psychological support, thereby enhancing patients' perceived family care and overall readiness.

#### 4.2.6 Living Arrangement

The study demonstrated that living status significantly associates ambulatory‐surgery patients discharge readiness; non‐solitary individuals exhibited higher readiness than those living alone, a finding consistent with Wang et al. (2021). Co‐residence with family or friends facilitates timely assistance with daily tasks and emotional support, whereas solitary living limits such resources. Consequently, nurses should intensify post‐discharge follow‐up for solitary patients, provide home visits when necessary and collaborate with community health services to ensure seamless transitional care.

#### 4.2.7 Occupation (Retirement)

In addition, retirement status was independently and negatively associated with discharge readiness, meaning retired patients had lower discharge readiness. In China, the statutory retirement age is 50–55 for women and 55–60 for men, so most retired individuals in this study were middle aged or elderly. Retired patients may face challenges like physical function decline, lower health literacy, weaker self‐management awareness and less social participation, which could reduce their ability to understand and implement discharge‐related regimens. This finding confirms the influence of age on discharge readiness. Therefore, more comprehensive, repeated and individualised discharge education should be provided to retired patients. The content should be simplified, family participation enhanced and follow‐up support strengthened to improve their post‐discharge recovery preparedness.

### 4.3 Limitations

This study has several limitations. First, this was a single‐centre study with a relatively small sample, which may limit the representativeness and generalisability of the findings. A multicentre design with a larger sample is warranted in future research. Second, the cross‐sectional design only allows identification of associations rather than causal relationships. Since discharge readiness and discharge teaching quality were assessed simultaneously at pre‐discharge, temporal ambiguity, same‐source bias and potential reverse causality cannot be avoided. Third, we did not use the C‐HOBIC scale for direct evaluation; future studies are suggested to combine theory‐based instruments with standardised scales such as C‐HOBIC to improve inter‐institutional comparability. Finally, the relatively high correlation between discharge teaching quality and discharge readiness may reflect conceptual overlap as well as common‐method bias, given that both were self‐reported by patients at the same time point, which should be considered in future investigations.

## 5 Summary and Prospects

This study identified multiple modifiable and non‐modifiable factors associated with discharge readiness among ambulatory surgery patients, covering personal, family, therapeutic and environmental dimensions. To optimise the hospital‐to‐home transition, clinical doctors and nurses should develop individualised, holistic discharge plans tailored to surgical specialty, age, living arrangement and retirement status. For modifiable factors including discharge teaching quality, health literacy and family support, targeted strategies are urgently needed: implementing standardised nurse training to improve teaching quality; providing patient‐centred education to enhance health literacy; and strengthening family engagement to reinforce social support. Simplified instructions, repeated education and strengthened post‐discharge follow‐up are also recommended to improve patients' self‐management ability and RHDS. These transitional care services led by nursing staff are of vital importance for the quality of patients' recovery.

To further advance transitional care, a systematic, technology‐enabled care framework should be established using integrated medical networks to connect hospital, community and home services seamlessly. Telehealth and artificial intelligence applications can be explored to meet the growing needs for high‐quality, continuous care. In future research, developing and validating specialised intervention programs is warranted to optimise discharge readiness and accelerate postoperative recovery. Meanwhile, integrating C‐HOBIC indicators into assessment is suggested to improve the accuracy of discharge readiness evaluation and achieve consistency with global standardised care transition metrics.

## 6 Conclusion

Discharge readiness of ambulatory‐surgery patients was moderate. Associated with personal, environmental, family and nursing therapeutic factors. Quality of discharge teaching, health literacy, surgical specialty (ophthalmology, otorhinolaryngology), age groups (45–59 and 75–89 years), family support, living arrangement and occupation (retirement) were independently associated with discharge readiness.

## Author Contributions

**Jing Zhang:** conceptualisation, methodology, data curation, data analysis, original draft and writing – review and editing. **Xiaoli Xing:** conceptualisation, methodology, supervision, writing – review. **Xiaodan Xia** and **Enjie Ping:** conceptualisation, research implementation, resources. **Xiufang Zhang** and **Jingjing Liu:** research implementation, writing – review. **Xiaorong Luan:** conceptualisation, methodology, writing – review.

## Funding

This research was supported by the following grants. Scientific Research Project of Wannan Medical College (Current Status and Influencing Factors of Discharge Readiness among Elderly Patients with Chronic Diseases, No. WK2023JXYY008), Huainan Science and Technology Plan Project (Development and Validation of a Patient Experience Assessment Tool for Ambulatory Surgery, No. 2021077), Anhui Provincial ‘13th Five‐Year’ Key Medical and Health Discipline Construction Project (Wan Wei Ke Jiao [2022] No. 30) and 2022 Anhui Provincial Clinical Key Specialty Construction Project (Wan Wei Yi Mi [2022] No. 105).

## Ethics Statement

This study was conducted in accordance with the Declaration of Helsinki and approved by the ethics committee of The First Hospital of Anhui University of Science and Technology (2022‐YJ‐004‐01).

## Consent

Written informed consent was obtained from all participants.

## Conflicts of Interest

The authors declare no conflicts of interest.

## Data Availability

The data that support the findings of this study are available from the corresponding author upon reasonable request.
